# Sleep Disturbances and the Speed of Multimorbidity Development in Old Age

**DOI:** 10.1093/geroni/igab046.1462

**Published:** 2021-12-17

**Authors:** Amaia Calderón-Larrañaga, Laura Pérez, Davide Vetrano, Federico Triolo, Linnea Sjöberg, Alexander Darin-Mattsson, Marco Inzitari, Shireen Sindi

**Affiliations:** 1 Karolinska Institutet, Solna, Stockholms Lan, Sweden; 2 Parc Sanitari Pere Virgili, Barcelona, Catalonia, Spain

## Abstract

Sleep disturbances are prevalent among older adults and are associated with various individual diseases. The goal of this study was to investigate whether sleep disturbances are associated with the speed of multimorbidity development among older adults. Data were gathered from the Swedish National study of Aging and Care in Kungsholmen (SNAC-K), an ongoing population-based study of subjects aged 60+ (N=3363). The study included a subsample (n=1189) without multimorbidity at baseline (<2 chronic diseases). Baseline sleep disturbances were assessed using the Comprehensive Psychiatric Rating Scale, and categorized as none, mild, moderate-severe. The number of chronic conditions throughout the nine-year follow-up was obtained from clinical examinations. Linear mixed models were used to study the association between sleep disturbances and the speed of chronic diseases accumulation, adjusting for sex, age, education, physical activity, smoking, alcohol consumption, depression, pain, and psychotropic drug use. We repeated the analyses including only cardiovascular, neuropsychiatric, and musculoskeletal diseases as the outcome. Moderate-severe sleep disturbances were associated with a higher speed of chronic disease accumulation (ß/year=0.142, p=0.008), regardless of potential confounders. Significant positive associations were also found between moderate-severe sleep disturbances and neuropsychiatric (ß/year=0.041, p=0.016) and musculoskeletal (ß/year=0.038, p=0.025) disease accumulation, but not with cardiovascular diseases. Results remained stable when participants with baseline dementia, cognitive impairment, or depression were excluded. The finding that sleep disturbances are associated with faster chronic disease accumulation points towards the importance of early detection and treatment of sleep disturbances as a possible strategy to reduce chronic multimorbidity among older adults.

